# Correlation Between Hepatic and Splenic Steatosis Assessed by Transient Elastography in Patients With Metabolic Dysfunction-Associated Steatotic Liver Disease

**DOI:** 10.7759/cureus.88856

**Published:** 2025-07-27

**Authors:** Abeer Altaf, Zaigham Abbas, Muhammad Ali Qadeer, Mehreen Siyal

**Affiliations:** 1 Gastroenterology, Dr. Ziauddin Hospital, Karachi, PAK; 2 Gastroenterology and Hepatology, Dr. Ziauddin Hospital, Karachi, PAK

**Keywords:** body mass index, chronic liver disease (cld), metabolic dysfunction-associated steatotic liver disease (masld), obesity, spleen steatosis

## Abstract

Objective

Metabolic dysfunction-associated steatotic liver disease (MASLD) can rapidly progress to steatohepatitis, liver fibrosis, and hepatocellular carcinoma. However, it is a multisystem disease, and most of the MASLD-related morbidity and mortality is due to its extrahepatic complications. In light of the growing impact of MASLD on extrahepatic organs, we aimed to evaluate spleen fat deposition and compare it with liver fat deposition by vibration-controlled transient elastography using its attenuation parameter.

Design

Eighty patients from the outpatient department, who met the criteria of MASLD, were included in the study. Both liver and spleen elastography were performed via FibroScan at the same time before treatment.

Results

Out of the 80 people studied, 69 (86.3%) were male patients. Their age ranged from 22 to 86 years (mean 42.44± 11.92), and BMI ranged between 20.5 to 39.8 kg/m^2^ (28.12± 3.99). Hypertension was present in 45 (56.3%) patients. Sixteen (20%) patients had mild liver steatosis, 26 (32.5%) had moderate, and 28 (35%) patients recorded the presence of severe fatty liver. Using the same cut-off values for spleen, 28 (35%) patients had normal attenuation (steatosis), four (5%) had mild spleen steatosis, 17 (21.3%) had moderate, and 31 (38.8%) patients had severe fatty spleen. A bivariate correlation analysis revealed a significant correlation between hepatic and splenic steatosis (p=0.01). Furthermore, patients with elevated spleen steatosis demonstrated higher liver fat content as indicated by raised liver controlled attenuation parameter (CAP) values (289.79±30.10 dB/m) compared to those without spleen steatosis (264.67±41.85 dB/m).

Conclusion

This study shows correlation of fatty liver with that of steatosis of the spleen, highlighting the extrahepatic multiorgan effects of the MASLD.

## Introduction

Nonalcoholic fatty liver disease (NAFLD), now renamed as metabolic dysfunction-associated steatotic liver disease (MASLD), is a term for steatotic liver disease associated with metabolic dysfunction [1]. Initially thought to be a disease of the West, its incidence in some regions of Asia has shown a dramatic increase of 40-50% [2]. In the past few years, MASLD has garnered attention from hepatologists due to its rising prevalence and its recognition as a progressive, severe liver disease. This is supported by data showing that new waitlist registrations for nonalcoholic steatohepatitis (NASH)-related cirrhosis increased by approximately 170% (from 804 to 2,174) between 2004 and 2013, demonstrating a sharp rise in liver transplant indications linked to NAFLD-related disease [3,4]. If things continue the way they are, it is predicted to become the most common indication for liver transplant by 2030 [5].

In recent years, it has been evident that MASLD’s public health burden is not just limited to liver-related pathologies. On the contrary, it is a multisystem disease, and most of MASLD morbidity and mortality is due to its extrahepatic complications (including cardiovascular disease, chronic kidney disease, type 2 diabetes, dyslipidemia, obstructive sleep apnea, and increased risk of certain cancers) [6]. Several studies have proven and investigated the pathophysiological link between MASLD and its associated conditions [7]. This established relationship has contributed to the development of improved diagnostic tools and emerging pharmacological therapies aimed at slowing the progression of MASLD.

Despite the close anatomical relationship of the spleen and the liver and MASLD’s proven extrahepatic complications, there are very limited studies conducted to find a relationship between ‘fatty spleen’ and ‘fatty liver’. A study conducted in Japan found that individuals with hepatic steatosis had more a spleen volume that was more than normal. They suspected that the increase in portosystemic pressure could be a possible cause of the increase in spleen volume, but there was very limited evidence to confirm this hypothesis [8]. Recent papers show a greater accuracy of spleen stiffness measurement while diagnosing clinically significant portal hypertension in MASLD [9]. However, these studies do not provide conclusive evidence regarding the clinical significance of elevated attenuation parameters in the spleen. Given the spleen’s role as an immunometabolic organ involved in lipid metabolism, systemic inflammation, and portal circulation, its potential involvement in MASLD warrants investigation beyond its anatomical relationship with the liver. This study aims to assess the correlation between hepatic and splenic steatosis using transient elastography, exploring whether spleen-based metrics may reflect or parallel liver fat accumulation in MASLD.

## Materials and methods

Study design and setting

This was a prospective cross-sectional study conducted at the outpatient clinics of the Department of Hepatogastroenterology at Dr. Zaiuddin Hospital, Karachi, Pakistan, between January 2022 and December 2023. The study received approval from the institutional ethics review board (Reference code: 4561221AAGE), and written informed consent was obtained from all participants before inclusion.

Study population

Inclusion Criteria

Adults aged ≥18 years

Clinically diagnosed with MASLD)

No known alternative etiology of chronic liver disease

No regular or excessive alcohol consumption in the past two years

Willing and able to provide informed consent

Exclusion Criteria

Active or past history of chronic hepatitis B or C

History of regular alcohol intake exceeding defined limits (>30 g/day in men, >20 g/day in women)

Decompensated liver disease (e.g., ascites, hepatic encephalopathy)

Pregnancy

History of liver transplantation

Presence of active implantable medical devices (e.g., pacemakers)

Clinically suspected genetic or metabolic liver diseases (e.g., Wilson's disease, hemochromatosis) were excluded based on clinical and biochemical profiles; liver biopsy and genetic testing were not routinely performed

Diagnostic criteria for MASLD

MASLD was diagnosed based on the evidence of hepatic steatosis on transient elastography (CAP ≥248 dB/m) in the presence of at least one of the following metabolic risk factors [10]:

Body mass index (BMI) ≥25 kg/m²

Type 2 diabetes mellitus

Hypertension or dyslipidemia

HOMA-IR >2.5

Patients with any other identifiable cause of liver disease were excluded based on their clinical history, baseline laboratory tests, and review of past medical records.

Data collection and variables

At enrollment, data were collected on the following:

Demographics: age, sex, BMI, waist circumference

Laboratory parameters: complete blood count, liver function tests, fasting blood sugar, fasting insulin, fasting lipid profile

Derived indices: Homeostatic Model Assessment of Insulin Resistance (HOMA-IR) calculated as fasting insulin (µU/mL) × fasting glucose (mg/dL)/405

A thorough clinical history and physical examination were conducted for each participant. Medical records were reviewed to ensure the exclusion of other liver disease etiologies.

Elastography measurements

All patients underwent transient elastography (FibroTouch®, Wuxi Hisky Medical Technologies Co., Ltd., China) using the M-probe. Liver stiffness measurements (LSM, in kilopascals (kPa)) and controlled attenuation parameter (CAP)0 values (in dB/m) were recorded. In the same session, splenic stiffness measurements were obtained using standardized splenic elastography protocol. All scans were performed by certified operators and interpreted by experienced hepatologists. Transient elastography measures stiffness (fibrosis) and attenuation (steatosis). The cut-off used for hepatic stiffness in this study, corresponding to the Metavir score, were: F0-F1 (<7 kPa), F2 (7-9.5 kPa), F3 (9.5-14 kPa), F4 (>14 kPa) [11]. The second component of attenuation, which signifies the fat accumulation, was measured as normal (<240 dB/m), mild (240-264 dB/m), moderate (265-294 dB/m), and severe (>295 dB/m) [12]. Ranges were kept similar for both spleen and liver as splenic cut-offs have not been validated.

Study outcomes

Primary outcome was the association between liver steatosis and splenic stiffness measurements. The secondary outcomes included the correlation of splenic stiffness with HOMA-IR, Aspartate Aminotransferase-to-Platelet Ratio Index (APRI), BMI, and other metabolic risk factors.

Sample size calculation

This was an exploratory, hypothesis-generating study. As no prior data were available on splenic attenuation in MASLD patients to inform effect size estimation, a formal sample size calculation based on a predefined endpoint was not feasible. Therefore, a sample size of 80 patients was selected based on logistical feasibility over the study duration, with the aim of generating initial estimates to guide future confirmatory studies.

Bias minimization

Selection bias was minimized through consecutive enrollment of eligible patients. Measurement bias was reduced by using standardized, validated elastography techniques performed by trained personnel. Laboratory analysis followed institutional protocols, and operators were blinded to clinical variables during imaging. Confounding factors were addressed in multivariate statistical models.

Statistical analysis

Data were analyzed using IBM SPSS Statistics for Windows, Version 25 (Released 2017; IBM Corp., Armonk, New York, United States). Continuous variables were reported as mean ± SD, and categorical variables as frequencies and percentages. Bivariate analysis was performed using Pearson's correlation to assess associations between clinical, laboratory, and elastography variables. Paired t-tests were used to compare liver and spleen stiffness and attenuation values across fibrosis and steatosis stages. Independent t-tests assessed the differences in liver attenuation between patients with and without splenic steatosis. A p-value <0.05 was considered statistically significant.

## Results

In this study, a total of 80 patients with MASLD were recruited, out of which 69 (86.3%) were male patients and 11 (13.75%) were female patients. Their ages ranged from 22 to 86 years (mean 42.44 ± 11.92), and BMI ranged between 20.5 to 39.8 kg/m^2^ (mean 28.12 ± 3.99 kg/m^2^). Hypertension was the most common comorbidity present in 45 patients (56.3%), followed by dyslipidemia in 43 (53.5%), diabetes in 17 (31.2%), and chronic liver disease in 11 (13.75%). Table 1 further summarizes the baseline laboratory values and transient elastography parameters of the study participants.

**Table 1:** Baseline characteristics of the study population HOMA-IR: Homeostatic Model Assessment for Insulin Resistance; ALT: Alanine aminotransferase; AST: Aspartate transaminase; ALP: Alkaline phosphatase; GGT: Gamma-glutamyl transferase Continuous variables are expressed as mean ± standard deviation (SD) with their respective units. Correlations were assessed using Pearson’s correlation coefficient (r) and associated p-values are reported where applicable.

Variable	Range (Min–Max)	Mean ± SD	P-value	Test statistic
Age (years)	22–86	42.44 ± 11.92	-	-
Weight (kg)	56–129	81.7 ± 13.5	-	-
Height (m)	1.52–1.87	1.7 ± 0.7	-	-
Body Mass Index (kg/m^2^)	20.5–39.8	28.12 ± 3.99	0.029	r = 0.246
Hemoglobin (g/dL)	9–17	14.12 ± 1.84	-	-
Total leucocyte count (x10⁹/L)	3.7–12.5	7.78 ± 1.94	-	-
Platelets (x10⁹/L)	55–434	244.53 ± 84.91	0.259	r= 0.147
ALT (U/L)	13–1382	86.13 ± 162.15	0.654	r= 0.055
AST (U/L)	10–1077	59.11 ± 133	0.788	r= 0.035
ALP (U/L)	38–276	97.93 ± 44.40	0.457	r= 0.091
GGT (U/L)	11–570	72.6 ± 90.69	-	-
Creatinine (mg/dL)	0.6–1.2	0.94 ± 0.15	0.020	r = -0.411
Fasting blood sugar (mg/dL)	80–214	104.61 ± 24.03	0.129	r= 0.190
Fasting insulin (mIU/L)	2–61	17.19 ± 11.04	0.673	r= 0.061
HOMA-IR			0.266	r= 0.159
Fasting triglycerides (mg/dL)	44–810	197.15 ± 120.96	0.926	r= -0.012
Fasting cholesterol (mg/dL)	104–264	179.92 ± 37.40	0.369	r= -0.115
Liver stiffness (kPa)	3–25.7	9.41 ± 5.070	0.535	r= 0.070
Liver attenuation (dB/m)	184–359	280.99 ± 36.45	0.001	r = 0.354
Splenic stiffness (kPa)	3–35.5	14.65 ± 7.36	0.227	r= -0.137
Spleen attenuation (dB/m)	188–381	277.91 ± 56.97	-	-

Liver attenuation values ranged from 184 to 359 with a mean of 280.99 ±36.45. Among the 80 patients, 16 (20%) had mild liver steatosis, 26 (32.5%) had moderate, and 28 (35%) suffered from severe hepatic steatosis. Similarly, in the case of spleen steatosis about four (5%) patients had mild, 17 (21.3%) had moderate, and 31 (38.8%) patients recorded severe steatosis on FibroScan (FibroTouch®, Wuxi Hisky Medical Technologies Co., Ltd., China). The elastography measurement of attenuation parameter, for fat content in the spleen, ranged from 188 to 381 with a mean of 277.77 ± 56.6 among the study participants.

A bivariate correlation analysis revealed a significant correlation between hepatic and splenic steatosis (p<0.05), BMI (p=0.029), and creatinine (p=0.020). Furthermore, an independent sample t-test demonstrated that patients with spleen steatosis had significantly higher liver attenuation values compared to those without steatosis (289.79 ± 30.10 vs 264.67 ± 41.85; t(78) = 3.10, p=0.003). Splenic stiffness and the attenuation values in relation to the liver values among the study population are given in Table 2.

**Table 2:** Paired comparison of liver and spleen stiffness (kPa) across fibrosis stages and attenuation (dB/m) across steatosis stages in the patients with MASLD (n=80) Values represent mean ± standard deviation. Mean differences, 95% confidence intervals (CI), and p-values were calculated using paired t-tests.

Stage	Measurement type	Liver (mean ± SD)	Spleen (mean ± SD)	Mean difference	95% CI	p-value
F0–F1	Stiffness (kPa)	5.71 ± 1.04	4.56 ± 1.48	+1.15	0.75 to 1.54	<0.001
F2	Stiffness (kPa)	8.91 ± 1.19	9.52 ± 1.52	–0.61	–1.03 to –0.19	0.006
F3	Stiffness (kPa)	11.85 ± 2.10	14.52 ± 3.77	–2.67	–3.62 to –1.72	<0.001
F4	Stiffness (kPa)	18.00 ± 4.41	19.31 ± 5.24	–1.31	–2.81 to 0.19	0.091
S1	Attenuation (dB/m)	254.68 ± 6.63	245.50 ± 6.36	+9.18	7.17 to 11.19	<0.001
S2	Attenuation (dB/m)	278.23 ± 7.58	276.81 ± 11.40	+1.42	–1.58 to 4.42	0.356
S3	Attenuation (dB/m)	314.53 ± 21.29	334.55 ± 32.88	–20.02	–28.60 to –11.44	<0.001

When paired t-tests were used to compare liver and spleen stiffness values within the same individuals, a significant trend was observed across the fibrosis stages in the 80 patients with MASLD. At F0-F1, the liver stiffness was significantly higher than spleen stiffness (mean difference: 1.15 kPa, 95% CI: 0.75 to 1.54, p<0.001). At F2, spleen stiffness exceeded liver stiffness (mean difference: -0.61 kPa, 95% CI: -1.03 to -0.19, p=0.006), and this difference widened at F3 (mean difference: -2.67 kPa, 95% CI: -3.62 to -1.72, p<0.001). At F4, the difference was not statistically significant (mean difference: -1.31 kPa, 95% CI: -2.81 to 0.19, p=0.091).

Similarly, paired t-tests comparing liver and spleen attenuation values across steatosis stages revealed a significant difference at S1 (mean difference: 9.18 dB/m, 95% CI: 7.17 to 11.19, p<0.001), no significant difference at S2 (mean difference: 1.42 dB/m, 95% CI: -1.58 to 4.42, p=0.356), and a reversal at S3, where spleen attenuation was significantly higher than the liver attenuation (mean difference: -20.02 dB/m, 95% CI: -28.6 to -11.44, p<0.001). These paired comparisons indicate a progressive elevation in spleen stiffness and attenuation in association with advancing liver fibrosis and steatosis, respectively.

## Discussion

Liver steatosis is simply an excessive accumulation of fat in the liver, eventually leading to inflammation and cirrhosis. It is often an overlooked silent pandemic and one of the leading causes of liver transplantation. It has now been widely understood that fatty liver also manifests as extrahepatic diseases such as cardiovascular diseases, chronic kidney disease, colorectal cancer, diabetes mellitus, and osteoporosis [13]. This has led to the coining of a new nomenclature for understanding as well as de-stigmatising fatty liver, bringing it under the umbrella term of MASLD [1].

Recent advances in the Baveno VII algorithm for correlating liver fibrosis with spleen stiffness as a reliable marker for predicting clinically significant portal hypertension, and its performance in ruling out high risk of varices [14], highlight the crucial role of the spleen in liver dysfunction. Through our study, we aimed to identify the relation between fat accumulation in the spleen as an extrahepatic manifestation of MASLD. We found a significant association between liver and splenic steatosis (CAP) and higher chances of fatty spleen in patients with moderate-to-severe hepatic lipid accumulation.

Several studies in the past have discussed the liver-spleen axis, especially in patients with MASLD, and its role in the possible immune cosplay responsible for orchestrating acute and eventually chronic inflammation [15]. Twenty-three years ago, Tsushima et al. [8] offered groundbreaking evidence of a correlation between spleen enlargement and hepatic fat content. Perhaps the most important role the spleen plays is to act as the chief immune organ of the human body. The primary pathophysiology of steatohepatitis is the tipping of the balance towards proinflammatory cytokines. Obesity reduces the level of IL-10 (an anti-inflammatory cytokine), and this, along with overproduction of inflammatory mediators such as tumor necrosis factor-α and IL-6, leads to progression of MASLD [16,17].

Our study also indicates increased splenic steatosis in patients with raised BMI (p=0.029). El-Aziz et al. previously noted that increased central obesity was correlated with increased splenic size seen on ultrasound [18], perhaps due to sinusoidal dilatation and raised intracellular lipid deposits in the spleen as a result of obesity. This finding was further corroborated by Gallagher et al. who found a reduction in the spleen size of patients with MASLD after weight loss [19]. Finally, the splenic enlargement in MASLD may be the result of portal hypertension secondary to ongoing chronic inflammation of liver, which may occur in MASLD even without cirrhosis. Yu et al. proposed the idea that increased splenic volume predicted higher chances of future decompensations in patients with compensated chronic liver disease [20].

The liver-spleen attenuation index or LAI is the difference between the average liver density and the average splenic density on a non-contrast CT scan [21]. It is used to assess the hepatic steatosis of possible living donors prior to liver transplantation. How accurate can the LAI be for donor compatibility in a patient who already has hepatic steatosis, given the potential of a fatty spleen? Which cutoff points can we utilize to determine a donor's lack of fitness for transplantation and to precisely quantify the level of "true" hepatic steatosis? How can we accurately estimate this ratio using the CAP of our transient elastography tools? These are only a handful of the unanswered questions raised by this study that demand more investigation.

Our study has several limitations. It was conducted at a single tertiary care center with a relatively small sample size, which may limit the generalizability of the findings. Histological validation or CT/MRI-based imaging of splenic fat was not performed due to ethical and feasibility constraints, and transient elastography-based splenic attenuation lacks established reference standards. Additionally, potential confounders such as physical activity, dietary intake, or systemic inflammatory markers were not evaluated. However, the study also has important strengths, including its prospective design, clearly defined diagnostic criteria, and use of a non-invasive, reproducible modality (transient elastography) to assess both the liver and spleen involvement. To our knowledge, this is among the first studies to explore splenic steatosis as an extrahepatic manifestation of MASLD, laying the groundwork for future research in this domain.

## Conclusions

This study demonstrates a significant association between hepatic steatosis and splenic steatosis in patients with MASLD, suggesting a potential “fatty spleen” phenomenon and further supporting the existence of a liver-spleen axis in metabolic liver disease. The use of non-invasive elastography enabled a novel insight into splenic involvement, particularly in patients with higher BMI and elevated creatinine. A key strength of this study is its prospective design and focus on an underexplored dimension of MASLD. However, the lack of histological confirmation and reliance on elastography-based cutoffs are notable limitations. Future studies should incorporate histopathological validation and larger, multicenter cohorts to better define splenic involvement and its prognostic value. Understanding this interplay may open new avenues for risk stratification and management in MASLD.
